# Numerical method to compute acoustic scattering effect of a moving source

**DOI:** 10.1186/s40064-016-3080-x

**Published:** 2016-08-24

**Authors:** Hao Song, Mingxu Yi, Jun Huang, Yalin Pan, Dawei Liu

**Affiliations:** School of Aeronautic Science and Engineering, Beihang University, Beijing, 100191 People’s Republic of China

**Keywords:** Ducted tail rotor, Aerodynamic characteristic, Aerodynamic noise, Acoustic velocity, Boundary element method

## Abstract

In this paper, the aerodynamic characteristic of a ducted tail rotor in hover has been numerically studied using CFD method. An analytical time domain formulation based on Ffowcs Williams–Hawkings (FW–H) equation is derived for the prediction of the acoustic velocity field and used as Neumann boundary condition on a rigid scattering surface. In order to predict the aerodynamic noise, a hybrid method combing computational aeroacoustics with an acoustic thin-body boundary element method has been proposed. The aerodynamic results and the calculated sound pressure levels (SPLs) are compared with the known method for validation. Simulation results show that the duct can change the value of SPLs and the sound directivity. Compared with the isolate tail rotor, the SPLs of the ducted tail rotor are smaller at certain azimuth.

## Background

In recent decades, noise pollution has become a major issue of concern and the noise generation mechanisms have been investigated widely. Noise generated by aircraft, fans and others has great influence on the aeroacoustic research (Polacsek et al. 1999; Greenwood and Schmitz 2014; Kingan 2014; Johnson 1980; Mao et al. 2015). In these applications, the direct sound field and scattering effect are always considered to assess the acoustic impact of sound sources (Mouille 1970, 1986; Lowson 2015).

Due to the effect of the duct, the slipstream pattern and aerodynamic characteristic of ducted tail rotor are different from the conventional open-type rotor. It can reduce the risk of component damage and enhance the operational safety. In addition, both the duct and rotor of the ducted tail rotor can produce thrust. Thrust which is produced by the duct is due to negative pressure on the duct inlet, which contributes a maximum of 50 % to the total. Furthermore, the noise of ducted tail rotor is lower than the conventional open-type rotor (Keys et al. 1991; Desjardins et al. 1991). Consequently, the analysis of the aerodynamic noise of the ducted tail rotor is the main topic for this paper.

In order to predict the aerodynamic noise, aerodynamic characteristics of the ducted tail rotor should be obtained first. Along with the development of the ducted tail rotor, many researchers have studied the aerodynamic characteristics of various types of ducted tail rotors (Rajagopalan and Keys 1997; Bandoh et al. 1998; Vuillet and Morelli 1986). In recent years, the main method which investigates the aerodynamic performance of ducted tail rotor is CFD (Computational Fluid Dynamic) method. Among those CFD methods, momentum source method is one of the commonly used methods. The momentum source with functional relationship to the local flow conditions represents the spanning rotor in flow field and are added to the N-S equations by UDF procedure. Cao and Yu (2005) used it to solve the numerical simulation of turbulent flow around ducted tail rotor. The method not only can predict the flow field but also investigate the performance of the ducted tail rotors. Song et al. (2009) applied it to analyze the aerodynamic characteristics of ducted tail rotor. To obtain the aerodynamic characteristics of the ducted tail rotor, the author solved the N-S equation added by the source. The numerical solutions were verified by the experimental data, and some aerodynamic characteristics of the ducted tail rotor were also discussed. Even though momentum source method is quite successful for the prediction of the aerodynamic performance of ducted tail rotor, detailed three dimensional flow features cannot be accurately predicted. Because of the approximation of the blade shape parameters, the accuracy of the momentum source method is still limited. In addition, it is difficult to obtain the accurate noise source information by using the momentum source method. In this work, a CFD method which is considered the blade shape parameters has been proposed to investigate the aerodynamic characteristics of ducted tail rotor, and the method is based on moving reference frame (MRF) model instead of momentum source method.

Calculation of the acoustic field radiated by rotating sources is a meaningful problem in the prediction of noise of aircraft rotors. The main methods for noise prediction are based on acoustic analogy Curle equation, FW–H (Ffowcs Williams–Hawkings) equation and the generalized treatment of Goldstein (Williams and Hawkings 1969; Farassat and Kenneth 1987, 1997). The inhomogeneous acoustic wave equation divides the aeroacoustic source into three types: monopole source, dipole source and quadrupole source.

Although the aerodynamic characteristics of ducted tail rotor have been discussed by many researchers (Cao et al. 2005; Yu and Cao 2006), the investigation of the aerodynamic noise is relatively few. The prediction of rotating blade aerodynamic noise can be acquired easily by solving FW-H equation, but the scattering effect of the duct wall on the propagation of the sound wave is difficult to discuss and the scattering mechanism is rather complex. The acoustic boundary element method (BEM) is usually applied to predict the sound radiating and scattering filed in the exterior and interior closed domain (Ih and Lee 1997; Wu 2000). Wu and Wan (1992) proposed a thin-body BEM, in which an imaginary interface is constructed to divide the domain into interior and exterior subdomains, and the imaginary surface is not discretized in the numerical implementation. The author indicated the scattering mechanism of the thin body boundary, and proposed the Helmholtz boundary integral equation to analyze the scattering effect.

In the present study, the analytical formulation of the acoustic velocity computation is also derived for sources in arbitrary motion. In order to consider the scattering effect of the thin duct, the newly developed FW–H–Helmholtz boundary elements method is introduced. The derived velocity analytical formulation is used as the Neumann boundary condition for the thin-body BEM. This method can predict the far-field aerodynamic noise of the ducted tail rotor.

## Governing equations

Given the constant rotary speed of the ducted tail rotor, the flow field in stable state is only taken into consideration to study its noise problems. So the unsteady Navier–Stokes equation is applied here to calculate the flow field. In rotating coordinate system, the unsteady Navier–Stokes equation can be expressed as follows (Li and Xiong 2007)

1 $$\frac{\partial }{\partial t}\iiint_{V} {WdV} + \iint_{\partial V} {H_{W} \cdot ndA} - \iint_{\partial V} {H_{V} \cdot ndA} + \iiint_{V} {S_{V} dV} = 0$$

where *V* is control body, ∂*V* is the boundary area, *n* is the normal vector. *W* is conservation vector,and $W{ = }\left[ {\rho ,\rho u_{0} ,\rho v_{0} ,\rho w_{0} ,\rho E} \right]^{T}$, *ρ* is the density of the fluid, *u*_0_, *v*_0_, *w*_0_ is projection of absolute velocity in relative coordinate, *E* is the internal energy of the unit fluid. *H*_*W*_ and *H*_*V*_ are the flux tensors. *S*_*V*_ is source term.

The flow field of the ducted tail rotor is acquired via solving the numerical solutions of Eq. (1). The standard *k* − *ɛ* model is adopted here because of its reasonable accuracy and fewer resources. The moving reference frame is also used to solve the problem of rotating. The far-field boundary is treated as the non-reflecting boundary condition. The detailed procedure for solving the governing equations can be found in Cao et al. (2003).

## Radiated sound field

### Ffowcs Williams–Hawkings equation

In 1969, Williams and Hawkings (1969) used the generalized function theory to derive the sound equation of the control plane in arbitrary motion in static fluid, that is, the famous FW–H equation. The FW–H equation is given by

2 $$\begin{aligned} & \left( {\frac{1}{{c^{2} }}\frac{\partial }{{\partial t^{2} }} - \frac{{\partial^{2} }}{{\partial x_{i}^{2} }}} \right)p'\left( {x,t} \right) = \frac{\partial }{\partial t}\left\{ {\left[ {\rho_{0} v_{n} + \rho \left( {u_{n} - v_{n} } \right)} \right]\delta \left( f \right)} \right\} \\ & \quad - \frac{\partial }{{\partial x_{i} }}\left\{ {\left[ { - P_{ij} n_{j} + \rho u_{i} \left( {u_{n} - v_{n} } \right)} \right]\delta \left( f \right)} \right\} + \frac{\partial }{{\partial x_{i} \partial x_{j} }}\left[ {T_{ij} H\left( f \right)} \right] \\ \end{aligned}$$

for inviscid flow, $\frac{1}{{c^{2} }}\frac{\partial }{{\partial t^{2} }} - \frac{{\partial^{2} }}{{\partial x_{i}^{2} }}$ is wave operator, *u*_*i*_ is velocity, *f* denotes a moving Kirchhoff surface, *p*’ is acoustic pressure, *v*_*n*_ is normal component of surface velocity. *P*_*ij*_ denotes the compressive stress tensor, $T_{ij} = - P_{ij} ' + \rho u_{i} u_{j} - c^{2} \rho '\delta_{ij}$ denotes a component of the Lighthill tensor, *δ*(*f*) is Dirac delta function, *H*(*f*) is Heaviside function and satisfied

$$H(f) = \left\{ {\begin{array}{*{20}c} 1 & {f\left( {x_{i} ,t} \right) > 0} \\ 0 & {f\left( {x_{i} ,t} \right) < 0} \\ \end{array} } \right., \quad \delta \left( f \right) = \frac{\partial H\left( f \right)}{\partial f}$$

As $f\left( {x_{i} ,t} \right) = 0$, according to non penetration condition, *u*_*n*_ = *v*_*n*_, FW–H equation can be reduced as below

3 $$\left( {\frac{1}{{c^{2} }}\frac{\partial }{{\partial t^{2} }} - \frac{{\partial^{2} }}{{\partial x_{i}^{2} }}} \right)p'\left( {x,t} \right) = \frac{\partial }{\partial t}\left[ {\rho_{0} v_{n} \delta \left( f \right)} \right] - \frac{\partial }{{\partial x_{i} }}\left[ {P_{ij} n_{j} \delta \left( f \right)} \right] + \frac{\partial }{{\partial x_{i} \partial x_{j} }}\left[ {T_{ij} H\left( f \right)} \right]$$

${{\partial \left[ {\rho_{0} v_{n} \delta (f)} \right]} \mathord{\left/ {\vphantom {{\partial \left[ {\rho_{0} v_{n} \delta (f)} \right]} {\partial t}}} \right. \kern-0pt} {\partial t}}$, $- \partial {{\left[ {P_{ij} n_{j} \delta (f)} \right]} \mathord{\left/ {\vphantom {{\left[ {P_{ij} n_{j} \delta (f)} \right]} {\partial x_{i} }}} \right. \kern-0pt} {\partial x_{i} }}$ and $\partial^{2} {{[T_{ij} H\text{(}f\text{)}]} \mathord{\left/ {\vphantom {{[T_{ij} H\text{(}f\text{)}]} {\partial x_{i} \partial x_{j} }}} \right. \kern-0pt} {\partial x_{i} \partial x_{j} }}$ are monopole source, dipole source and quadruple source, respectively. According to Hanson and Fink’s theory, Eq. (3) can be simplified to

4 $$\left( {\frac{1}{{c^{2} }}\frac{\partial }{{\partial t^{2} }} - \frac{{\partial^{2} }}{{\partial x_{i}^{2} }}} \right)p^{\prime}(x\text{,}\;t) = \frac{\partial }{\partial t}\left[ {\rho_{0} v_{n} \delta (f)} \right] - \frac{\partial }{{\partial x_{i} }}\left[ {P_{ij} n_{j} \delta (f)} \right]$$

### Farassat method

During the 1970’s and the 1980’s, based on the integral of Green Function, conversion of the spatial derivatives and time derivatives, Farassat published the famous Farassat 1 and Farassat 1A formulations which are the solution of time domain integral expressions for the thickness noise and loading noise of FW–H equation. The solution of Eq. (4), the formulation of Farassat 1A, is expressed as follows (Farassat and Kenneth 1987)

5 $$p^{\prime}(x,t) = p^{\prime}_{T} (x,t) + p^{\prime}_{L} (x,t)$$

with

6 $$4\pi p^{\prime}_{T} (x,t) = \int_{S} {\left[ {\frac{{\rho_{0} \dot{v}_{n} }}{{r(1 - M_{r} )^{2} }}} \right]}_{ret} dS + \int_{S} {\left[ {\frac{{\rho_{0} v_{n} (r\dot{M}_{i} \hat{r}_{i} + c_{0} M_{r} - c_{0} M^{2} )}}{{r^{2} (1 - M_{r} )^{3} }}} \right]}_{ret} dS$$

7 $$\begin{aligned} 4\pi p^{\prime}_{L} (x,t) & = \frac{1}{{c_{0} }}\int_{S} {\left[ {\frac{{\dot{l}_{i} \hat{r}_{i} }}{{r(1 - M_{r} )^{2} }}} \right]}_{ret} dS + \int_{S} {\left[ {\frac{{l_{r} - l_{i} M_{i} }}{{r^{2} (1 - M_{r} )^{2} }}} \right]}_{ret} dS \\ & \quad + \frac{1}{{c_{0} }}\int_{S} {\left[ {\frac{{l_{r} (r\dot{M}_{i} \hat{r}_{i} + c_{0} M_{r} - c_{0} M^{2} )}}{{r^{2} (1 - M_{r} )^{3} }}} \right]}_{ret} dS \\ \end{aligned}$$

$p^{\prime}_{T} (x,t)$ is thickness noise and $p^{\prime}_{L} (x,t)$ is loading noise. The subscript *ret* indicates that all of the values have to be taken at the retarded time. The dot over the quantity denotes the differentiation of this magnitude with respect to the emission time.

## Acoustic velocity formulation for sources in arbitrary motion

The procedure of the velocity formulation for the thickness and loading sources has been recently proposed by (Farassat 2007). Following the same procedure gives the thickness and loading acoustic velocity as follows:

8 $$4\pi \rho_{0} a^{\prime}_{Ti} (x,t) = - \int_{S} {\frac{\partial }{{\partial x_{i} }}\left[ {\frac{Q}{{r(1 - M_{r} )}}} \right]}_{ret} dS$$

9 $$4\pi \rho_{0} a^{\prime}_{Li} (x,t) = - \frac{1}{{c_{0} }}\int_{S} {\frac{\partial }{{\partial x_{i} }}\left[ {\frac{{L_{r} }}{{r(1 - M_{r} )}}} \right]}_{ret} dS - \int_{0}^{t} {\left( {\int_{S} {\frac{\partial }{{\partial x_{i} }}\left[ {\frac{{L_{r} }}{{r^{2} (1 - M_{r} )}}} \right]_{{ret^{*} }} } dS} \right)dt^{*} }$$

where $Q = \rho (u_{n} - v_{n} ) + \rho_{0} v_{n}$, $a^{\prime}_{Ti}$ and $a^{\prime}_{Li}$ are the acoustic velocity components for the thickness and loading sources. *u*_*n*_ and *v*_*n*_ are the fluid and the data surface normal velocity, respectively. *ρ* denotes the local fluid density, and *ρ*_0_ is the density of the undisturbed medium. The subscript *ret* ^*^indicates that all of the values have to be taken at the retarded time *t* ^*^.

For any function *F*(*x*, *τ*(*x*, *t*)), one has

10 $$\left. {\frac{\partial F}{{\partial x_{i} }}} \right|_{t} = \left. {\frac{\partial F}{{\partial x_{i} }}} \right|_{\tau } + \left. {\frac{\partial F}{\partial \tau }} \right|_{x} \left. {\frac{\partial \tau }{{\partial x_{i} }}} \right|_{t}$$

Based on Eq. (10) and $\left. {\frac{\partial \tau }{\partial t}} \right|_{x} = \left[ {\frac{1}{{(1 - M_{r} )}}} \right]_{ret}$, the velocity formulation V1 for the thickness and loading sources can be obtained as follows

11 $$4\pi \rho_{0} a^{\prime}_{Ti} (x,t) = \frac{1}{{c_{0} }}\frac{\partial }{\partial t}\int_{S} {\left[ {\frac{{Q\hat{r}_{i} }}{{r(1 - M_{r} )}}} \right]}_{ret} dS + \int_{S} {\left[ {\frac{{Q\hat{r}_{i} }}{{r^{2} (1 - M_{r} )}}} \right]}_{ret} dS$$

12 $$\begin{aligned} 4\pi \rho_{0} a^{\prime}_{Li} (x,t) & = \frac{1}{{c_{0}^{2} }}\frac{\partial }{\partial t}\int_{S} {\left[ {\frac{{\hat{r}_{i} L_{r} }}{{r(1 - M_{r} )}}} \right]}_{ret} dS - \frac{1}{{c_{0} }}\int_{S} {\left[ {\frac{{L_{i} - 3\hat{r}_{i} L_{r} }}{{r^{2} (1 - M_{r} )}}} \right]}_{ret} dS \\ & \quad - \int_{0}^{t} {\left( {\int_{S} {\left[ {\frac{{L_{i} - 3\hat{r}_{i} L_{r} }}{{r^{3} (1 - M_{r} )}}} \right]_{{ret^{*} }} } dS} \right)dt^{*} } \\ \end{aligned}$$

To improve the speed and accuracy of the velocity formulation V1, the time derivatives of formulation V1 can be taken inside the integrals. From the Eq. (11), one obtains

13 $$\frac{\partial }{\partial t}\int_{S} {\left[ {\frac{{Q\hat{r}_{i} }}{{r\left( {1 - M_{r} } \right)}}} \right]_{ret} dS = } \int_{S} {\left[ {\hat{r}_{i} \frac{\partial }{\partial t}\left( {\frac{Q}{{r\left( {1 - M_{r} } \right)}}} \right)} \right]_{ret} dS + } \int_{S} {\left[ {\frac{Q}{{r\left( {1 - M_{r} } \right)}}\frac{{\partial \hat{r}_{i} }}{\partial t}} \right]_{ret} dS}$$

Since

14 $$\left. {\frac{{\partial \left( {\hat{r}_{i} } \right)_{ret} }}{\partial t}} \right|_{x} = \left[ {\frac{1}{{r\left( {1 - M_{r} } \right)}}} \right]_{ret} \left. {\frac{{\partial \left( {\hat{r}_{i} } \right)_{ret} }}{{\partial \tau_{ret} }}} \right|_{x} - \left[ {\frac{{r_{i} }}{{r^{2} \left( {1 - M_{r} } \right)}}} \right]_{ret} \left. {\frac{{\partial r_{ret} }}{{\partial \tau_{ret} }}} \right|_{x} = \left[ {\frac{{ - c_{0} M_{i} + c_{0} \hat{r}_{i} M_{r} }}{{r\left( {1 - M_{r} } \right)}}} \right]_{ret}$$

The second term of the right hand of Eq. (13) can be given by

15 $$\int_{S} {\left[ {\frac{Q}{{r\left( {1 - M_{r} } \right)}}\frac{{\partial \hat{r}_{i} }}{\partial t}} \right]_{ret} dS} = c_{0} \int_{S} {\left[ {Q\frac{{M_{r} \hat{r}_{i} - M_{i} }}{{r^{2} \left( {1 - M_{r} } \right)^{2} }}} \right]_{ret} dS}$$

Then Eq. (11) becomes

16 $$\begin{aligned} 4\pi \rho_{0} a^{\prime}_{Ti} \left( {x,t} \right) & = \frac{1}{{c_{0} }}\int_{S} {\left[ {\hat{r}_{i} \frac{\partial }{\partial t}\left( {\frac{Q}{{r\left( {1 - M_{r} } \right)}}} \right)} \right]_{ret} dS} \\ & \quad & + \,\int_{S} {\left[ {Q\frac{{M_{r} \hat{r}_{i} - M_{i} }}{{r^{2} \left( {1 - M_{r} } \right)^{2} }}} \right]_{ret} dS + } \int_{S} {\left[ {\frac{{Q\hat{r}_{i} }}{{r^{2} \left( {1 - M_{r} } \right)}}} \right]_{ret} dS} \\ \end{aligned}$$

or

17 $$4\pi \rho_{0} a^{\prime}_{Ti} \left( {x,t} \right) = \frac{1}{{c_{0} }}\int_{S} {\left[ {\hat{r}_{i} \frac{\partial }{\partial t}\left( {\frac{Q}{{r\left( {1 - M_{r} } \right)}}} \right)} \right]_{ret} dS - } \int_{S} {\left[ {Q\frac{{M_{i} - \hat{r}_{i} }}{{r^{2} \left( {1 - M_{r} } \right)^{2} }}} \right]_{ret} dS}$$

Let

18 $$I_{T} = \frac{\partial }{\partial t}\left[ {\frac{Q}{{r(1 - M_{r} )}}} \right] = \frac{1}{{1 - M_{r} }}\frac{\partial }{\partial \tau }\left[ {\frac{Q}{{r(1 - M_{r} )}}} \right]$$

Expansion of the expression in the same manner as in the derivation of Farassat 1A gives

19 $$I_{T} = \frac{{\dot{Q}}}{{r(1 - M_{r} )^{2} }} + Q\frac{{r\dot{M}_{r} + c_{0} (M_{r} - M^{2} )}}{{r^{2} (1 - M_{r} )^{3} }}$$

where $\dot{M}_{r} = \dot{M}_{i} \hat{r}_{i}$. Then the acoustic velocity components for the thickness sources are got

20 $$4\pi \rho_{0} a^{\prime}_{Ti} (x,t) = \frac{1}{{c_{0} }}\int_{S} {\left[ {\hat{r}_{i} I_{T} } \right]}_{ret} dS - \int_{S} {\left[ {Q\frac{{M_{i} - \hat{r}_{i} }}{{r^{2} (1 - M_{r} )^{2} }}} \right]}_{ret} dS$$

Next, simplifying Eq. (12) further, one can rewrite it as follows

21 $$\begin{aligned} 4\pi \rho_{0} a^{\prime}_{Li} \left( {x,t} \right) & = \frac{1}{{c_{0} }}\int_{S} {\hat{r}_{i} \frac{\partial }{\partial t}\left[ {\frac{{L_{r} }}{{r\left( {1 - M_{r} } \right)}}} \right]_{ret} dS + \frac{1}{{c_{0}^{2} }}\int_{S} {\left[ {\frac{{L_{r} }}{{r(1 - M_{r} )}}\frac{{\partial \hat{r}_{i} }}{\partial t}} \right]_{ret} } dS} \\ & \quad - \frac{1}{{c_{0} }}\int_{S} {\left[ {\frac{{L_{i} - 3\hat{r}_{i} L_{r} }}{{r^{2} \left( {1 - M_{r} } \right)}}} \right]_{ret} dS - } \int_{0}^{t} {\left( {\int_{S} {\left[ {\frac{{L_{i} - 3\hat{r}_{i} L_{r} }}{{r^{3} \left( {1 - M_{r} } \right)}}} \right]_{{ret^{*} }} dS} } \right)dt^{*} } \\ \end{aligned}$$

Using Eq. (14), then

22 $$\begin{aligned} 4\pi \rho_{0} a^{\prime}_{Li} \left( {x,t} \right) & = \frac{1}{{c_{0} }}\int_{S} {\left[ {\hat{r}_{i} \left( {\frac{1}{{c_{0} }}\frac{\partial }{\partial t}\left[ {\frac{{L_{r} }}{{r\left( {1 - M_{r} } \right)}}} \right]_{e} + \left[ {\frac{{L_{r} }}{{r^{2} \left( {1 - M_{r} } \right)}}} \right]} \right)} \right]_{ret} dS} \\ & \quad - \frac{1}{{c_{0} }}\int_{S} {\left[ {L_{r} \frac{{M_{i} - \hat{r}_{i} }}{{r^{2} \left( {1 - M_{r} } \right)^{2} }}} \right]_{ret} dS - } \frac{1}{{c_{0} }}\int_{S} {\left[ {\frac{{L_{i} - L_{r} \hat{r}_{i} }}{{r^{2} \left( {1 - M_{r} } \right)}}} \right]_{ret} dS} - \int_{0}^{t} {\left( {\int_{S} {\left[ {\frac{{L_{i} - 3\hat{r}_{i} L_{r} }}{{r^{3} \left( {1 - M_{r} } \right)}}} \right]_{{ret^{*} }} dS} } \right)dt^{*} } \\ \end{aligned}$$

Let

23 $$I_{L} = \frac{1}{{c_{0} }}\frac{\partial }{\partial t}\left[ {\frac{{L_{r} }}{{r(1 - M_{r} )}}} \right]_{e} + \left[ {\frac{{L_{r} }}{{r^{2} (1 - M_{r} )}}} \right]$$

Expansion of the expression in the same manner as in the derivation of Farassat 1A gives

24 $$I_{L} = \frac{1}{{c_{0} }}\frac{{L_{r} }}{{r(1 - M_{r} )^{2} }} + \frac{{L_{r} - L_{M} }}{{r^{2} (1 - M_{r} )^{2} }} + \frac{1}{{c_{0} }}L_{r} \frac{{r\dot{M}_{r} + c_{0} (M_{r} - M^{2} )}}{{r^{3} (1 - M_{r} )^{3} }}$$

where $\dot{L}_{r}$ = $\dot{L}_{i} \mathop {r_{i} }\limits^{ \wedge }$, *L*_*M*_ = *L*_*i*_ *M*_*i*_. Finally the acoustic velocity components for the loading sources are obtained as follows

25 $$\begin{aligned} 4\pi \rho_{0} a^{\prime}_{Li} (x,t) & = \frac{1}{{c_{0} }}\int_{S} {\left[ {\hat{r}_{i} I_{L} } \right]_{ret} } dS - \frac{1}{{c_{0} }}\int_{S} {\left[ {L_{r} \frac{{M_{i} - \hat{r}_{i} }}{{r^{2} (1 - M_{r} )^{2} }}} \right]}_{ret} dS \\ & \quad - \frac{1}{{c_{0} }}\int_{S} {\left[ {\frac{{L_{i} - \hat{r}_{i} L_{r} }}{{r^{2} (1 - M_{r} )}}} \right]}_{ret} dS - \int_{0}^{t} {\left( {\int_{S} {\left[ {\frac{{L_{i} - 3\hat{r}_{i} L_{r} }}{{r^{3} (1 - M_{r} )}}} \right]_{{ret^{*} }} } dS} \right)dt^{*} } \\ \end{aligned}$$

**Fig. 1:**
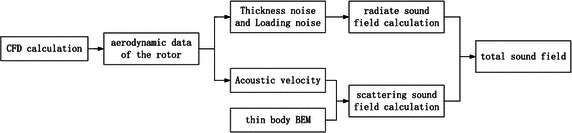
A flowchart of the total sound field calculation

A brief flowchart is shown in Fig. 1 to connect the relationship between sections “Governing equations”, “Radiated sound field” and “Acoustic velocity formulation for sources in arbitrary motion”. The flowchart also explains the whole numerical procedure of the total sound field.

## Thin-body acoustic boundary element method (BEM)

**Fig. 2:**
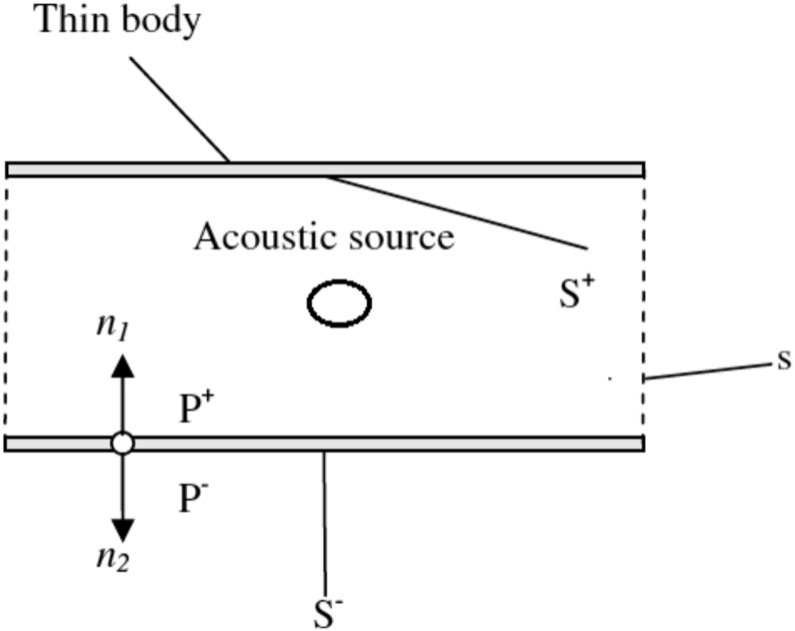
A diagram of acoustic scattering by a thin-body

In this section, a thin-body boundary integral formulation is applied to calculate the far field sound pressure. Due to the scattering effect of the solid wall in the duct, the total sound pressure is acquired as the sum of the incident and scattered pressure

26 $$P^{\prime}(x,\omega ) = P^{\prime}_{I} (x,\omega ) + P^{\prime}_{S} (x,\omega )$$

where $P^{\prime}_{I}$ and $P^{\prime}_{S}$ are incident and scattered sound pressure in the frequency domain, respectively. Based on Eqs. (6), (7), the incident sound pressure can be obtained and was transformed into data in frequency domain via using Fast Fourier Transform (FFT) method. The scattered sound pressure will be got by using BEM. The calculated domain is shown in Fig. 2, the surface of the duct is denoted as *S*, an imaginary surface *s*is added to close the duct and divide the computational domain into an interior subdomain *D* ^+^ and an exterior subdomain *D* ^−^. The sound pressure on the outside of the surface *S* + *s* is denoted by $P^{{{\prime } - }}$ and that on the inside is denoted by $P^{{{\prime } + }}$. The integral equation can be used to each subdomain (Wu and Wan 1992)

27 $$C^{ + } (x)P^{{{\prime } + }} (x,\omega ) = P_{I}^{{\prime }} (x,\omega ) + \int_{S + s} {\left[ {\frac{{\partial P^{{{\prime } + }} (y,\omega )}}{{\partial n_{1} (y)}}G(x,y,\omega ) - P^{{{\prime } + }} (y,\omega )\frac{\partial G(x,y,\omega )}{{\partial n_{1} (y)}}} \right]} dS(y)$$

28 $$C^{ - } (x)P^{{{\prime } - }} (x,\omega ) = \int_{S + s} {\left[ {\frac{{\partial P^{{{\prime } - }} (y,\omega )}}{{\partial n_{2} (y)}}G(x,y,\omega ) - P^{{{\prime } - }} (y,\omega )\frac{\partial G(x,y,\omega )}{{\partial n_{2} (y)}}} \right]} dS(y)$$

where *n*_1_ and *n*_2_ are normal unit vectors at the two sides of the wall, $C^{ + } (x)$ and $C^{ - } (x)$ are the two constants that depend on the position of *x*

29 $$C^{ + } (x) = \left\{ \begin{array}{ll} 0, &\quad x \in D^{ - } \\ 1, &\quad x \in D^{ + } \\ 1/2, &\quad x \in S \hfill \\ \end{array} \right.$$

30 $$C^{ - } (x) = \left\{ \begin{array}{ll} 0,& x \in D^{ + } \\ 1, &\quad x \in D^{ - } \\ 1/2,&\quad x \in S \\ \end{array} \right.$$

When *x* ∊ *S*, $C^{ + } (x) = C^{ - } (x) = \frac{1}{2}$. Therefore, adding Eqs. (27) and (28) gives the thin-body boundary integral equation

31 $$\begin{aligned} & \frac{1}{2}[P^{{{\prime } + }} (x,\omega ) + P^{{{\prime } - }} (x,\omega )] = P_{I}^{{\prime }} (x,\omega ) \\ & \quad + \int_{S} {\left\{ {G(x,y,\omega )\left[ {\frac{{\partial P^{{{\prime } + }} (y,\omega )}}{\partial n(y)} - \frac{{\partial P^{{{\prime } - }} (y,\omega )}}{\partial n(y)}} \right]} \right.} \\ & \quad \left. { - \frac{\partial G(x,y,\omega )}{\partial n(y)}[P^{{{\prime } + }} (y,\omega ) - P^{{{\prime } - }} (y,\omega )]} \right\}dS(y) \\ \end{aligned}$$

When $x \notin S$, adding Eqs. (27) and (28), then

32 $$P^{\prime}(x,\omega ) = P_{I}^{\prime } (x,\omega ) + \int_{S} {\left\{ {G(x,y,\omega )\left[ {\frac{{\partial P^{\prime + } (y,\omega )}}{\partial n(y)} - \frac{{\partial P^{\prime - } (y,\omega )}}{\partial n(y)}} \right]} \right.} \left. { - \frac{\partial G(x,y,\omega )}{\partial n(y)}[P^{\prime + } (y,\omega ) - P^{\prime - } (y,\omega )]} \right\}dS(y)$$

where $\partial /\partial n_{1} = - \partial /\partial n_{2} = \partial /\partial n$, and the continuous boundary conditions of the pressure and its partial derivation on the imaginary surface *s* are used

33 $$P^{{{\prime } + }} (x,\omega ) = P^{{{\prime } - }} (x,\omega ),\;\;\;\frac{{\partial P^{{{\prime } + }} (x,\omega )}}{\partial n(x)} = \frac{{\partial P^{{{\prime } - }} (x,\omega )}}{\partial n(x)},\;\;\;x \in s$$

The assumption of acoustic rigid boundary conditions are applied over the entire surface *S*

34 $$\frac{{\partial P^{{{\prime } + }} (x,\omega )}}{\partial n(x)} = \frac{{\partial P^{{{\prime } - }} (x,\omega )}}{\partial n(x)} = 0,\;\;\;x \in S$$

Then Eqs. (31) and (32) reduce to

35 $$\begin{aligned} & \frac{1}{2}[P^{{{\prime } + }} (x,\omega ) + P^{{{\prime } - }} (x,\omega )] = P_{I}^{{\prime }} (x,\omega ) \\ & \quad - \int_{S} {\left\{ {\frac{\partial G(x,y,\omega )}{\partial n(y)}[P^{{{\prime } + }} (y,\omega ) - P^{{{\prime } - }} (y,\omega )]} \right\}} dS(y),\;\;x \in S \\ \end{aligned}$$

36 $$P^{{\prime }} (x,\omega ) = P_{I}^{{\prime }} (x,\omega ) - \int_{S} {\left\{ {\frac{\partial G(x,y,\omega )}{\partial n(y)}[P^{{{\prime } + }} (y,\omega ) - P^{{{\prime } - }} (y,\omega )]} \right\}} dS(y),\;\;\;\;x \in D^{ + } \cup D^{ - } \cup s$$

Equation (36) is not sufficient to obtain the two unknowns $P^{{{\prime } + }} (x,\omega )$ and $P^{{{\prime } - }} (x,\omega )$. Differentiating Eq. (35) with regard to the direction of normal vector*n*(*x*), it can be transformed into

37 $$0 = \frac{{\partial P_{I}^{{\prime }} (x,\omega )}}{\partial n(x)} - \int_{S} {\left\{ {\frac{{\partial^{2} G(x,y,\omega )}}{\partial n(y)\partial n(x)}[P^{{{\prime } + }} (y,\omega ) - P^{{{\prime } - }} (y,\omega )]} \right\}} dS(y),\;\;x \in S$$

The problem of scattering by the duct can be dealt with, by initially solving Eq. (37) to calculate the sound pressure jump $P^{{{\prime } + }} (y,\omega ) - P^{{{\prime } - }} (y,\omega )$ on the surface *S*, and afterwards evaluating the acoustic pressure at any filed point. Then the acoustic pressure on both sides of the duct could be easily got. The value of $\frac{{\partial P_{I}^{{\prime }} (x,\omega )}}{\partial n(x)}$ cannot be obtained easily via using Eqs. (6) or (7), but can be acquired it indirectly by using the acoustic velocity formulation. If Eq. (37) satisfies the Neumann boundary condition, then

38 $$\frac{{\partial P_{I}^{{\prime }} (x,\omega )}}{\partial n(x)} = - i\omega \rho_{0} v_{n} (x,\omega )$$

where $v_{n} = \left\{ {a_{T1}^{{\prime }} (x,\omega ),\;a_{T2}^{{\prime }} (x,\omega ),a_{T3}^{{\prime }} (x,\omega )} \right\}$ for the thickness sources, $v_{n} = \left\{ {a_{L1}^{{\prime }} (x,\omega ),\;a_{L2}^{{\prime }} (x,\omega ),a_{L3}^{{\prime }} (x,\omega )} \right\}$ for the loading sources.

Then, to solve the Eq. (37), a discretized scheme based on BEM should be used to calculate the unknown value $P^{{{\prime } + }} (y,\omega ) - P^{{{\prime } - }} (y,\omega )$. The simplest constant boundary element is applied in this paper and the thin-body surface *S* is discretized into *N* elements. Each element has one node which is located in the center of the element. Then, Eq. (37) can be transformed into a system of algebraic equations.

39 $$\phi_{i} = \sum\limits_{j = 1}^{N} {\left( {A_{j} \cdot dS_{j} } \right) \cdot \varphi_{j} } = \sum\limits_{j = 1}^{N} {B_{j} \cdot \varphi_{j} } ,\;\;\;\;\;\;\;\;(i = 1, \ldots ,N)$$

where $\phi_{i} = - i\omega \rho_{0} v_{n} (x_{i} ,\omega )$, $A_{j} = \frac{{\partial^{2} G(x_{i} ,y_{j} ,\omega )}}{{\partial n(y_{j} )\partial n(x_{i} )}}$ and $\varphi_{j} = P^{{{\prime } + }} (y_{j} ,\omega ) - P^{{{\prime } - }} (y_{j} ,\omega )$, *x*_*i*_ and *y*_*j*_ are the center of element *i*, *j*, respectively. *dS*_*j*_ is the area of the element *j*. Therefore, the form of matrix of Eq. (39) is written as

40 $$\text{[}\varvec{\phi}\text{]}^{T} = [\varvec{B}][\varvec{\varphi }]^{T}$$

Equation (40) may be solved easily by using the software Mathematica. When the unknown $\varvec{\varphi }$ is calculated, the acoustic pressure at any filed point can be obtained through Eqs. (35) and (36).

The function *G*(*x*, *y*, *ω*) and its derivations $\frac{\partial G(x,y,\omega )}{\partial n(y)}$, $\frac{{\partial^{2} G(x,y,\omega )}}{\partial n(x)\partial n(y)}$ in above equations are expressed as follows:

41 $$G(x,y,\omega ) = \frac{{e^{jkr} }}{4\pi r}$$

42 $$\frac{\partial G(x,y,\omega )}{\partial n(y)} = - \left( {jk + \frac{1}{r}} \right)\frac{{e^{jkr} }}{4\pi r}\frac{\partial r}{\partial n(y)}$$

43 $$\frac{{\partial^{2} G(x,y,\omega )}}{\partial n(x)\partial n(y)} = \frac{{e^{jkr} }}{{4\pi r^{3} }}[( - 2 + 2jkr + k^{2} r^{2} )] \times n(x) \cdot n(y)$$

## Numerical results and discussions

In this section, the sound pressure is expressed as dB (decibels) and the predicted SPLs (sound pressure levels) is given by the following

44 $$SPLs = 20\lg \frac{{P_{e} }}{{P_{r} }}$$

where *P*_*e*_ denotes predicted pressure, *P*_*r*_ denotes the reference pressure and equals to 2 × 10^−5^Pa.

### Pulsating sphere

**Fig. 3:**
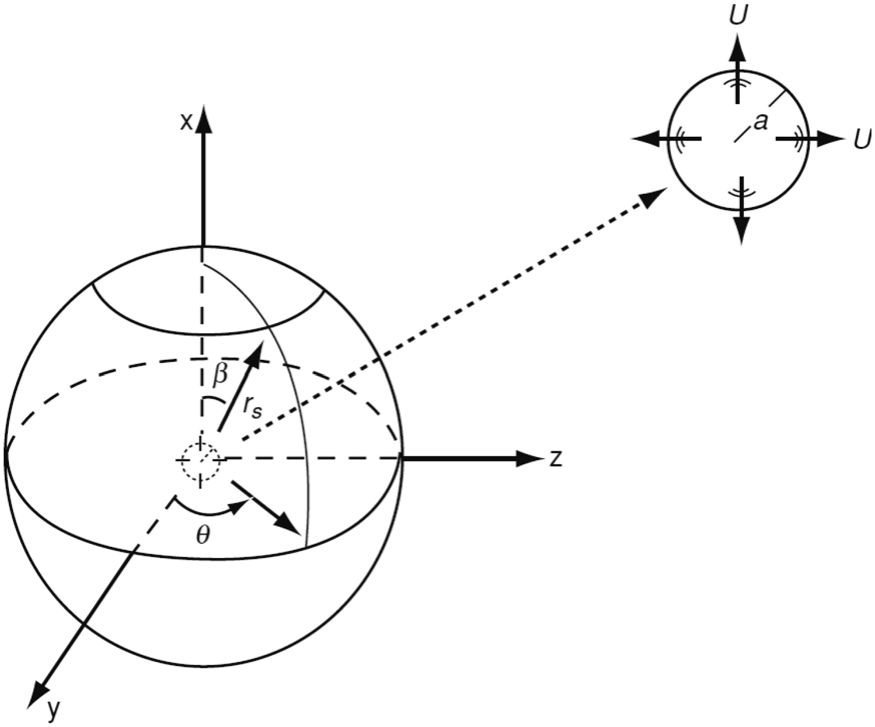
Monopole source and data surface are shown

In order to verify the algorithms, the analytical solution of a monopole source has been investigated. The monopole is identified with a pulsating sphere as the small sphere with a radius *a* in Fig. 3. The pressure fluctuation induced by the pulsating sphere is expressed by a harmonic spherical wave

45 $$p^{{\prime }} = \frac{{A\omega \rho_{0} }}{4\pi r}\frac{1}{{\sqrt {1 + \left( {ka} \right)^{2} } }}\cos \left[ {\omega t - k\left( {r - a} \right) + \phi_{0} } \right]$$

where *ω* and *k* are angular velocity and the wave number, respectively, and

46 $$\phi_{0} = \tan^{ - 1} \left( {\frac{1}{ka}} \right),\quad r = \sqrt {x^{2} + y^{2} + z^{2} }$$

**Fig. 4:**
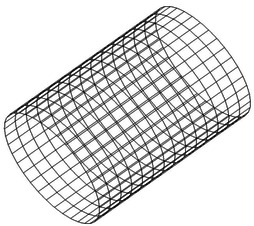
The duct in BEM

The velocity is given by

47 $$u_{r} \left( {r,t} \right) = \frac{1}{{\sqrt {1 + \left( {ka} \right)^{2} } }}\left( {\frac{Ak}{4\pi r}\cos \left[ {\omega t - k\left( {r - a} \right) + \varphi_{0} } \right]\left. { + \frac{A}{{4\pi r^{2} }}\sin \left[ {\omega t - k\left( {r - a} \right) + \varphi_{0} } \right]} \right)} \right.$$

where $A = 4\pi a^{2} U$, the radius of the spherical penetrable data surface *r*_*s*_ equals to 3.25*a*. The speed of sound *c*_0_is 340 m/s. The density for medium is 1.2 kg/m^3^. The angular velocity of the source is 1020 rad/s. The other parameters are *a* = 0.01 m and *U* = 8 m/s. The pulsating sphere is located at the center of the duct, which is shown in Fig. 4. The diameter of the duct is 0.07 m. The length of the duct is 0.5 m. The observer distance is assumed to be 1 m.

**Fig. 5:**
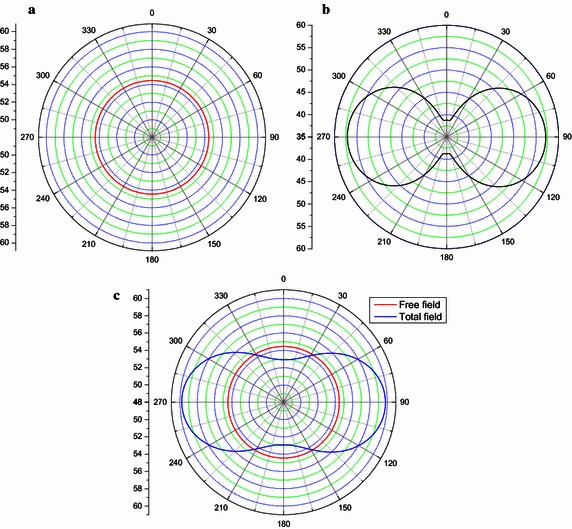
Directivity of calculated far-field SPLs at 4918 Hz **a** free field, **b** scattering effect of the duct and **c** total field

The thin-body BEM is used to perform the acoustic scattering problems of the duct. And the acoustic velocity is calculated by using Eq. (20) and used as Neumann boundary condition for this problem. Figure 5 shows the scattering performance of the pulsating sphere. The left is the incident sound pressure, the middle is the scattering effect of the duct and the right is the total sound pressure.

From the Fig. 4, it is possible to see that the directivity of the incident sound pressure is circular as the property of monopole sound source. When the scattering effect is considered, the directivity of the sum sound becomes non-circular. For the angle (210°–330° and 30°–150°), SPLs of the total field is louder due to scattering where at the angle (−30° to 30° and 150° to 210°), it is quieter. It shows that the sound pressure is strengthened in the direction of duct both ends, and the sound pressure is subdued in the other direction.

### Ducted tail rotor

#### Geometric configuration and mesh generation

**Fig. 6:**
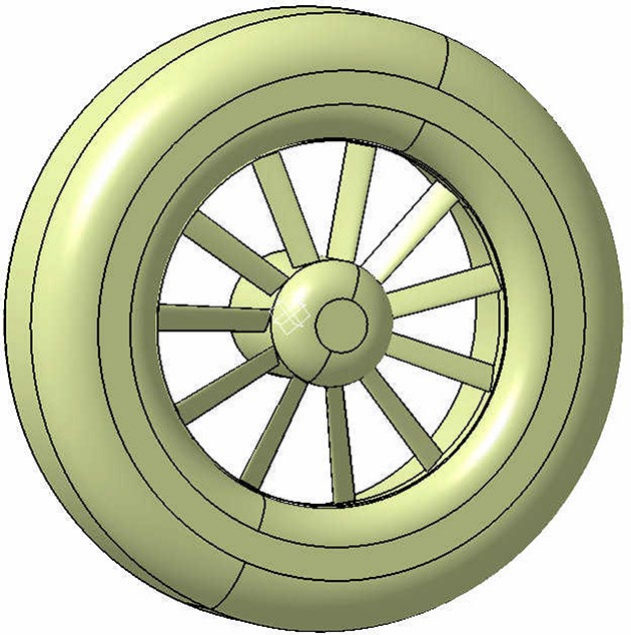
Geometric model of TsAGI ducted tail rotor

The model of TsAGI ducted tail rotor, as shown in Fig. 6, is used to demonstrate the effectiveness of the present method. It has been installed on the Ka-60 helicopter. Its geometric dimensions are tabulated in Table 1 as reported in the Bourtsev and Selemenev (2000).

**Table 1:** Geometric dimensions of the ducted tail rotor

*Rotor*	
Radius	297 mm
Number of blades	11
Solidity	0.4951
Twist angle	−12°
Tip speed	74.6 m/s
Airfoil	NACA23012 (r/R = 0.35 ~ 1.0)
*Duct*	
Tip clearance gap	0.01R
Inlet radius	0.2R
Diffuser length	0.7R
Diffuser angle	4°

**Fig. 7:**
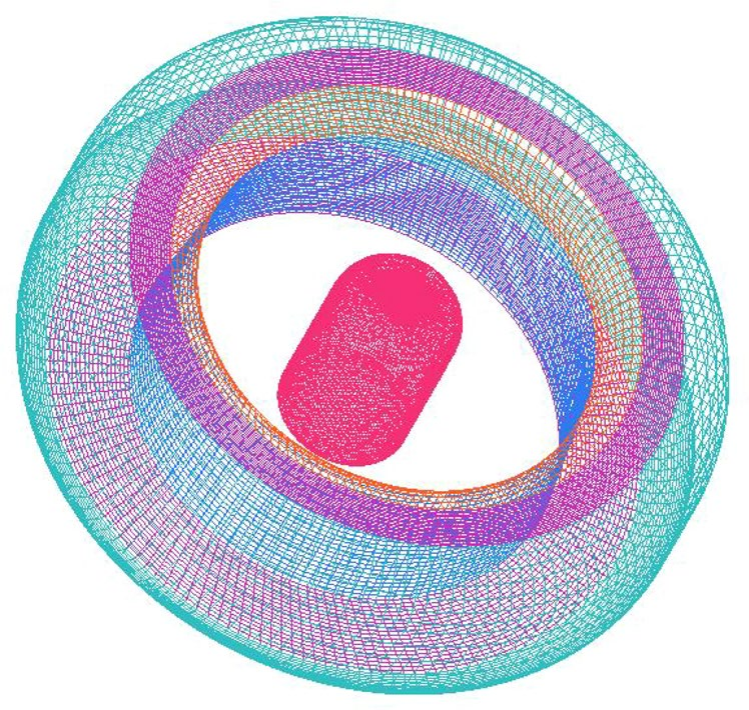
Surface quadrilateral for the duct

**Fig. 8:**
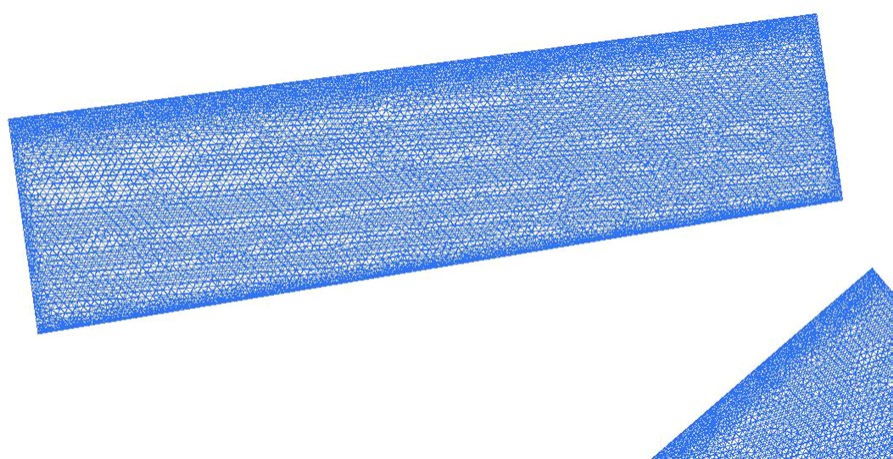
Surface quadrilateral for the tail rotor

**Fig. 9:**
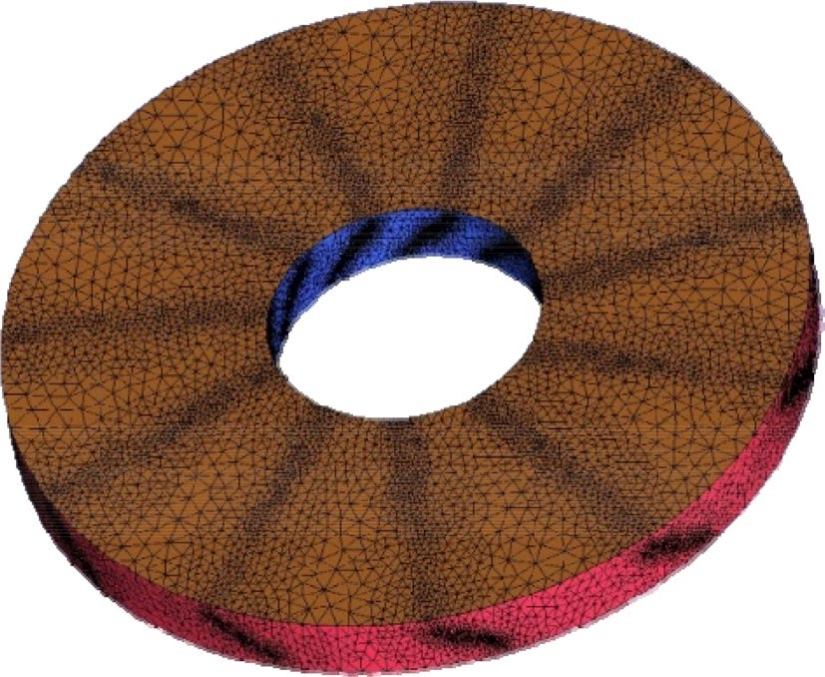
The unstructured grid of the rotating area

**Fig. 10:**
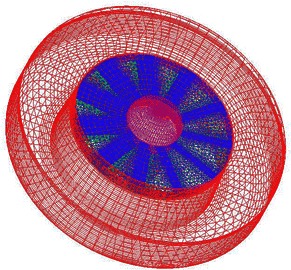
The overall mesh of the ducted tail rotor

A structured mesh is generated around the duct of the TsAGI model, and the surface quadrilateral for the all configuration is presented in Fig. 7. The number of the structured grid cell is 153 k. The tail rotor of the TsAGI model is meshed with an unstructured mesh which is shown in Fig. 8, and an adaptive encryption method is adopted to mesh the leading edge and the trailing edge of the tail rotor. During the calculation, the tail rotor is involved in a rotating area which is meshed with an unstructured grid (See Fig. 9). The number of the unstructured grid cell is 343 k. So the total number of the computational grid is 496 k. Figure 10 shows the overall mesh of the ducted tail rotor.

#### Aerodynamic performance of the ducted tail rotor

An initial condition is made for the ducted rail rotor at a tip mach number of 0.22 and a collective pitch angle of 40° at the blade root. At this moment, the predicted thrust from the present calculation is 9.547 kg. The rotor thrust in the Tan et al. (2008) and Lee and Kwon (2003) are 9.54 kg and 9.69 kg respectively. The coincidence indicates that our method can investigate aerodynamic performance of ducted tail rotor effectively.

**Fig. 11:**
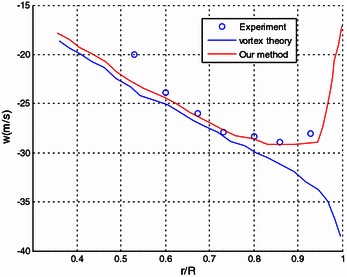
Comparison of the spanwise distribution of the induced downwash

**Fig. 12:**
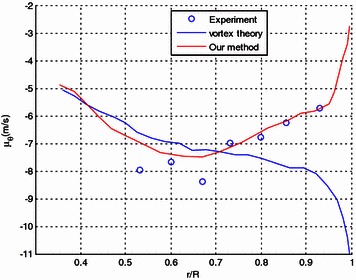
Comparison of the spanwise distribution of the circumferential induced velocity

In Figs. 11, 12, spanwise distribution of the induced downwash and the circumferential induced velocity are compared with the experiment and the results obtained by vortex theory (Wu 2000). It is also shown that the induced downwash and the circumferential induced velocity are generally in good coherence with the experiment results. The results obtained from our method also compare well with that of the vortex theory.

**Fig. 13:**
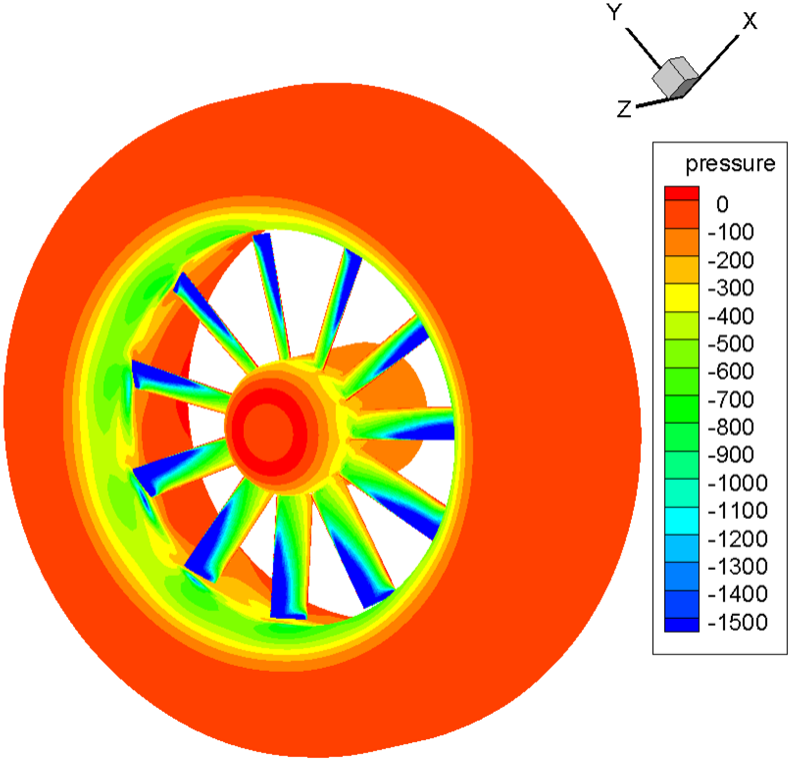
Pressure distribution for the surface of the ducted tail rotor

Figure 13 displays the pressure variation on the surface of the ducted tail rotor. As can be seen in Fig. 13, the pressure changes violently on the surface of the rotor and the duct lip, and others remain the same. Furthermore, the conclusion can be reached from Fig. 13: the thrust of ducted tail rotor is generated mainly by the rotors and the duct lip.

A grid sensitivity analysis is shown for the CFD simulation to illustrate the convergence of the method. The induced downwash and the circumferential induced velocity are discussed for the hybrid grids by increasing the meshing parameters. Their mean error (*e*_*mean*_) and maximum error (*e*_max_) are shown in Table 2, where *D* denotes the max grid cell size.

**Table 2:** Mean and maximum values of induced downwash and velocity for different grid densities

Meshing parameters		Induced downwash		Circumferential induced velocity	
Grid cells (k)	*D*(mm)	*e*_*mean*_	*e*_max_	*e*_*mean*_	*e*_max_
82	2.024	1.035	4.101	0.815	1.584
102	1.326	0.893	3.538	0.771	1.359
211	1.078	0.887	2.998	0.514	1.115
496	0.864	0.777	2.634	0.371	0.969
688	0.631	0.630	2.204	0.223	0.838
861	0.517	0.436	1.969	0.158	0.775
932	0.203	0.257	1.431	0.103	0.516

Form the Table 2, it is possible to find that the mean and maximum errors for the flow variables are smaller and smaller with the grid cells increasing. The small errors also show a converging trend.

#### Aerodynamic noise of the ducted tail rotor

**Fig. 14:**
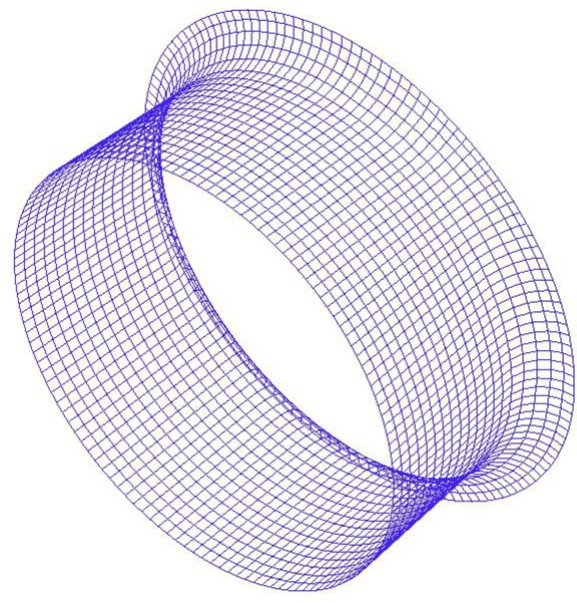
Mesh of duct used in BEM

The unsteady flow properties and the enough pressure data of the surface of the rotor can be acquired via the analysis of aerodynamic performance of TsAGI ducted tail rotor. Then they are transformed into data in frequency domain by applying fast Fourier transform method. The far- filed sound pressure is calculated using FW-H equation in the frequency domain. The acoustic velocity is calculated by using Eqs. (20), (25) and used as Neumann boundary condition. Before using the BEM method to study the radiation and propagation of the sound source, a simplified configuration, as shown in Fig. 14, should be used to consider the scattering effect of the duct. In this section, for a low speed rotor application, it is shown that dipole source is dominant and monopole source and quadrupole source shown to be negligible.

**Fig. 15:**
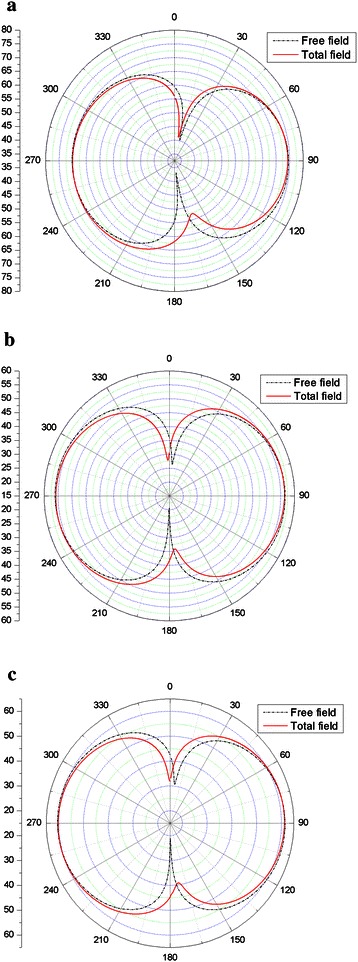
Noise directivity pattern at 440 Hz: **a** *r* = 1 *m*; **b** *r* = 5 *m*; **c** *r* = 3 *m*

**Fig. 16:**
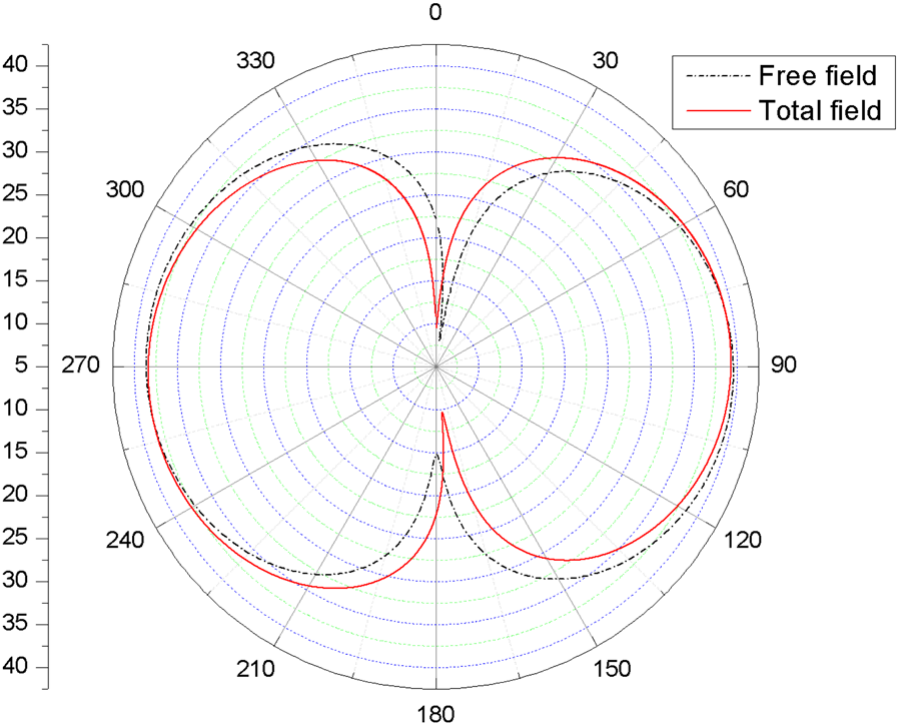
Noise directivity pattern at 880 Hz: *r* = 3 *m*

Acoustic pressure in frequency domain is calculated by combing FW-H equation and the thin- body BEM. For different observation distance, the directivity of SPLs at 440 Hz and 880 Hz are shown in Figs. 15 and 16 respectively. Free field is also rotor alone. The more interesting result is the comparison of the total field and the free field. This provides information about the scattering effect of the duct. For the angle (180°–360°), upstream of the rotor (180°–270°) is slightly louder due to scattering where at the angle (270°–360°), it is quieter. From the Fig. 15a, it is easy to see that the sound pressure at inlet is bigger than that of the outlet. From the Figs. 15b, c and 16, it is possible to find that the directivity of the incident sound pressure is symmetrical as the properties of the dipole sound source.

In order to illustrate the validation of the scheme, the results obtained in this work are compared with the results obtained by commercial software Virtual. Lab. Acoustics (VLA) (Zhan and Xu 2013). VLA is commercial simulation software which used specially to noise analysis. It can approximately analyze the aerodynamic noise and vibration noise by using boundary element method and finite element method. The comparisons are shown in 17.

**Fig. 17:**
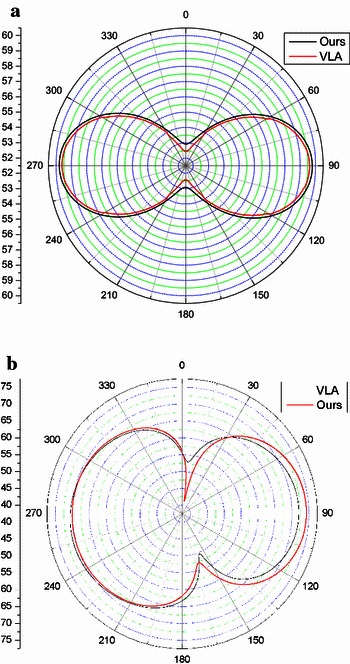
The comparison of the SPLs for different method: **a** Monopole source; **b** Ducted tail rotor

In Fig. 17, the SPLs obtained by our method are in good coincidence with those obtained by using VLA.

## Conclusions

The purpose of this work is to propose the FW-H/thin-body BEM method for predicting the aerodynamic noise of the ducted tail rotor in hover numerically. Based on the CFD method, aerodynamic characteristic of the TsAGI ducted tail rotor are discussed, and the results are compared with vortex theory and momentum source method for validation. The numerical method is convergent by increasing the number of the grid. The thrust of the duct is generated by duct lip.

A thin-body BEM is developed to predict the far-field aerodynamic noise of the ducted tail rotor. An analytical formulation is derived for prediction the acoustic velocity generated by moving bodies. It can be used as boundary condition for the thin-body BEM. The scattering effect of the pulsating sphere and ducted tail rotor are investigated by using the thin-body BEM. The calculated SPLs accord well with the results obtained by VLA, and show that our method effectively predict the far-field SPLs of the ducted tail rotor. Numerical examples show that the duct can change the value of SPLs and the sound directivity both the pulsating sphere and tail rotor. Furthermore, there are some contributed works including the scattering effect of high speed impulsive noise and the experimental verification for the numerical method of noise prediction.

## List of symbols

*a*_*Li*_^′^: Acoustic velocity components for loading source
*a*_*Ti*_^′^: Acoustic velocity components for thickness source
∂*V*: Boundary area
*T*_*ij*_: Component of the Lighthill tensor
*P*_*ij*_: Compressive stress tensor
*V*: Control body
*W*: Conservation vector
*v*_*i*_: Data surface velocity
*r*: Distance between observer and source
*r*: Distance between the source and the receiver
*δ*(*f*): Dirac delta function
*S*: Duct surface
*ρ*: Fluid density
*u*_*i*_: Fluid velocity
*H*_*W*_, *H*_*V*_: Flux tensors
*G*(*x*, *y*, *ω*): Green function in frequency domain
*G*(*x*, *y*, *t* − *τ*): Green function in time domain
*H*(*f*): Heaviside function
*P*_*I*_^′^: Incident sound pressure in frequency domain
*s*: Imaginary surface
*E*: Internal energy of the unit fluid
*f*: Kirchhoff surface
*p*_*L*_^′^: Loading noise
*l*_*i*_: Loading source term
*v*_*n*_: Normal component of surface velocity
*n*(*x*): Normal unit vector on the observer surface
*n*(*y*): Normal unit vector on the source surface
*n*: Normal vector
*x*: Observer position
*t*: Observe time
*P*_*e*_: Predicted pressure
*u*_0_, *v*_0_, *w*_0_: Projection of absolute velocity in relative coordinate
*P*_*r*_: Reference pressure
*ω*: Rotational angular velocity
*M*: Source Mach number vector
*M*_*r*_: Source Mach number in radiation direction, $M_{i} \hat{r}_{i}$
*P*^′^: Sound pressure in frequency domain
*P*_*S*_^′^: Scattering sound pressure in frequency domain
*p*^′^: Sound pressure in time domain
*c*_0_: Sound speed
*y*: Source position
*S*_*V*_: Source term
*τ*: Source time
*p*_*T*_^′^: Thickness noise
*k*: Wave number
