# Erratum to: Numerical method to compute acoustic scattering effect of a moving source

**DOI:** 10.1186/s40064-017-3790-8

**Published:** 2017-05-31

**Authors:** Hao Song, Mingxu Yi, Jun Huang, Yalin Pan, Dawei Liu

**Affiliations:** School of Aeronautic Science and Engineering, Beihang University, Beijing, 100191 People’s Republic of China

## Erratum to: SpringerPlus (2016) 5:1404 DOI 10.1186/s40064-016-3080-x

The description of “innovation” and “references” in our article (Song et al. 2016) needs additional clarification.

Firstly, in the paper “Acoustic velocity formulation for sources in arbitrary motion” (Ghorbaniasl et al. 2013) the theory and the method are particularly good and valuable, and its general method is not only applied to their proposed fields, but is also good at handling the scattering effects appearing in the aviation field.

Secondly, we (Song et al. 2016) studied the theory and method described by Ghorbaniasl et al. (2013) and found they are very suitable for reducing the scattering effect of noise. In the aviation field, the prediction problem of acoustic noise of the ducted tail rotor is very important and difficult to solve; therefore, we tried to use their method to solve the problem. According to our simulation, the perfect results of the acoustic noise of the ducted tail rotor are acquired. To the best of our knowledge, this is the first attempt to predict the acoustic noise of the ducted tail rotor using the theory and the method proposed by Ghorbaniasl et al. (2013).

Thirdly, we omitted to cite three articles (Ghorbaniasl et al. 2013; Mao et al. 2008; Hu et al. 2013). We apologize to readers and to the authors of these articles. We therefore present the following corrections:

1. The procedure of the velocity formulation for the thickness and loading sources was proposed by Farassat (2007). Following the same procedure gives the thickness and loading acoustic velocity as follows (Ghorbaniasl et al. 2013):

8 $$ 4\pi \rho_{0} a_{Ti}^{\prime } (x,t) = - \int_{S} {\frac{\partial }{{\partial x_{i} }}\left[ {\frac{Q}{{r(1 - M_{r} )}}} \right]}_{ret} dS $$

9 $$ 4\pi \rho_{0} a_{Li}^{\prime } (x,t) = - \frac{1}{{c_{0} }}\int_{S} {\frac{\partial }{{\partial x_{i} }}\left[ {\frac{{L_{r} }}{{r(1 - M_{r} )}}} \right]}_{ret} dS - \int_{0}^{t} {\left( {\int_{S} {\frac{\partial }{{\partial x_{i} }}\left[ {\frac{{L_{r} }}{{r^{2} (1 - M_{r} )}}} \right]_{{ret^{*} }} } dS} \right)dt^{*} } $$

2. Next, simplifying Eq. (12) further, one can rewrite it as follows (Ghorbaniasl et al. 2013):

21 $$ \begin{aligned} 4\pi \rho_{0} a_{Li}^{{\prime }} \left( {x,t} \right) & = \frac{1}{{c_{0} }}\int_{S} {\hat{r}_{i} \frac{\partial }{\partial t}\left[ {\frac{{L_{r} }}{{r\left( {1 - M_{r} } \right)}}} \right]_{ret} dS + \frac{1}{{c_{0}^{2} }}\int_{S} {\left[ {\frac{{L_{r} }}{{r(1 - M_{r} )}}\frac{{\partial \hat{r}_{i} }}{\partial t}} \right]_{ret} } dS} \\ & \quad - \frac{1}{{c_{0} }}\int_{S} {\left[ {\frac{{L_{i} - 3\hat{r}_{i} L_{r} }}{{r^{2} \left( {1 - M_{r} } \right)}}} \right]_{ret} dS - } \int_{0}^{t} {\left( {\int_{S} {\left[ {\frac{{L_{i} - 3\hat{r}_{i} L_{r} }}{{r^{3} \left( {1 - M_{r} } \right)}}} \right]_{{ret^{*} }} dS} } \right)dt^{*} } \\ \end{aligned} $$

3. The sound pressure on the outside of the surface $S + s$ is denoted by $P^{{{\prime } - }}$ and that on the inside is denoted by $P^{{{\prime } + }}$. The integral equation can be used to each subdomain (Wu and Wan 1992; Mao et al. 2008; Hu et al. 2013)

27 $$ C^{ + } (x)P^{\prime + } (x,\omega ) = P_{I}^{\prime } (x,\omega ) + \int_{S + s} {\left[ {\frac{{\partial P^{\prime + } (y,\omega )}}{{\partial n_{1} (y)}}G(x,y,\omega ) - P^{\prime + } (y,\omega )\frac{\partial G(x,y,\omega )}{{\partial n_{1} (y)}}} \right]} dS(y) $$

28 $$ C^{ - } (x)P^{\prime - } (x,\omega ) = \int_{S + s} {\left[ {\frac{{\partial P^{\prime - } (y,\omega )}}{{\partial n_{2} (y)}}G(x,y,\omega ) - P^{\prime - } (y,\omega )\frac{\partial G(x,y,\omega )}{{\partial n_{2} (y)}}} \right]} dS(y) $$
